# Optimization of an immunostaining protocol for the rapid intraoperative evaluation of melanoma sentinel lymph node imprint smears with the 'MCW melanoma cocktail'

**DOI:** 10.1186/1742-6413-1-2

**Published:** 2004-08-06

**Authors:** Vinod B Shidham, Richard Komorowski, Virgilia Macias, Sushma Kaul, Glen Dawson, William W Dzwierzynski

**Affiliations:** 1 Department of Pathology, Medical College of Wisconsin, Milwaukee, WI, USA; 2 Department of Plastic Surgery, Medical College of Wisconsin, Milwaukee, WI, USA

**Keywords:** Immunocytochemistry, Melanoma, Sentinel lymph node, MCW melanoma cocktail, Melan- A, MART- 1, Tyrosinase, Intraoperative cytology, Micrometastasis, Lymphadenectomy

## Abstract

**Background:**

In the management of cutaneous melanoma, it is desirable to complete the regional lymphadenectomy during the initial surgical procedure for wide excision of biopsy site and sentinel lymph node (SLN) biopsy. In this study, we optimized and evaluated a rapid 17 minutes immunostaining protocol. The discriminatory immunostaining pattern associated with the 'MCW Melanoma Cocktail' (mixture of Melan- A, MART- 1, and tyrosinase) facilitated the feasibility of intraoperative evaluation of imprint smears of SLNs for melanoma metastases.

**Methods:**

Imprint smears of 51 lymph nodes from 25 cases (48 SLNs and 3 non-SLNs, 1 to 4 SLNs/case) of cutaneous melanoma were evaluated.

**Results:**

Sixteen percent, 8/51 lymph nodes (28%, 7/25 cases) were positive for melanoma metastases in immunostained permanent sections with the 'MCW melanoma cocktail'. All of these melanoma metastases, except 1 SLN from 1 case, were also detected in rapidly immunostained wet-fixed and air-dried smears (rehydrated in saline and postfixed in alcoholic formalin). The cytomorphology was superior in air-dried smears, which were rehydrated in saline and postfixed in alcoholic formalin. Wet-fixed smears frequently showed air-drying artifacts, which lead to the focal loss of immunostaining. None of the 5 SLNs from 5 cases exhibiting capsular nevi showed a false positive result with immunostained imprint smears.

**Conclusions:**

Melanoma metastases can be detected intraoperatively in both air-dried smears and wet-fixed smears immunostained with the MCW Melanoma cocktail. Air-dried smears rehydrated in saline and postfixed in alcoholic formalin provide superior results and many practical benefits.

## Background

### What is already known on this topic?

A rapid intraoperative evaluation of sentinel lymph nodes (SLNs) for melanoma metastases during the interval between the SLN biopsy and the wide excision of the melanoma biopsy site may eliminate the need of an additional surgery for completion of regional lymphadenectomy.

### What this study adds?

Air-dried imprint smears which were postfixed in alcoholic formalin following saline rehydration were optimal for immunocytochemical evaluation with the 'MCW melanoma cocktail'. The rapid evaluation of imprint smears immunostained with the 'MCW melanoma cocktail' is reliable for the intraoperative evaluation of cutaneous melanoma SLNs for melanoma metastases.

The prevailing trend in the management of cutaneous melanoma supports the sentinel lymph node (SLN) biopsy as a standard of care [1-16], but a few authors regard it as controversial [17,18]. In a given case where the SLN biopsy is performed and is positive for melanoma metastases, it is usually followed by additional surgery for regional lymphadenectomy. A rapid intraoperative evaluation of SLNs for melanoma metastases during the interval between the SLN biopsy and the wide excision of the melanoma biopsy site may eliminate the need for an additional regional lymphadenectomy surgery. Previously evaluated approaches such as fluorodeoxyglucose-positron emission tomography [19,20], morphological evaluation of frozen sections [21-24], intraoperative morphological evaluation of imprint cytology [25,26], and immunostaining of frozen sections [27] are not sufficiently sensitive.

Imprint smears of lymph nodes can be prepared rapidly. When compared to frozen sectioning, imprint smears are more desirable due to lower cost, quicker process, avoidance of tissue loss in the cryostat, prevention of freezing artifact in the tissue, and the elimination of problems associated with cryo-sectioning of fatty lymph nodes. These advantages have resulted in a preference for imprint smears over frozen sections by many investigators for the evaluation of SLNs in breast carcinoma [28,29].

Although relatively specific, the morphological interpretation of imprint smears alone used for the evaluation of melanoma metastases in SLNs is not very sensitive [25]. This is predominantly because of the inherent limitations associated with morphological interpretation. Singly scattered cells of melanoma metastases in a sea of numerous other cells are difficult to differentiate from reactive histiocytes, endothelial cells, and other cells with morphology alone.

At the current time, frozen-section examination (with or without immunohistochemical evaluation) and the morphological evaluation of imprint cytology smears are the methods available for intraoperative evaluation of SLN in cutaneous melanoma. However, these studies have demonstrated relatively low sensitivity and specificity, discouraging the practical application [22-27].

Conventional immunomarkers such as the S-100 protein and HMB45 suffer a significant drawback because of interference by non-melanoma cells resulting in high signal to noise ratio [28]. Because of this, rapid and accurate intraoperative evaluation of SLN with immunostained imprint smears was not previously possible.

In our previous study, the 'MCW Melanoma Cocktail'- a mixture of monoclonal antibodies- MART-1 {1:500}, Melan- A {1:100}, and Tyrosinase {1:50} (Table 1) demonstrated a highly discriminatory immunostaining pattern [28]. This observation suggested the feasibility of rapid intraoperative evaluation by examining the imprint smears of SLNs immunostained with the cocktail [28-30]. In the current study, we have optimized a protocol for the rapid intraoperative immunostaining of SLN imprint smears from patients with cutaneous melanoma utilizing the 'MCW melanoma cocktail'. Our previous experience suggested that air-dried smears postfixed in alcoholic formalin after saline rehydration demonstrated optimal results for immunostaining [31]. In this study, in addition to air-dried smears we also evaluated wet-fixed smears for further confirmation.

**Table 1:** The composition of the 'MCW Melanoma Cocktail'^¶^

Marker	Clone	Source	*Final Dilution in the cocktail
MART-1	M2-7C10	Signet Laboratories, Inc. Dedham, MA	1:500
Melan-A	A103	Dako Corporation, Carpinteria, CA	1:100
Tyrosinase	T311	Novocastra Laboratories Ltd Newcastle upon Tyne, UK	1:50

* Optimum dilution for each antibody was standardized individually for that batch of antibodies with the sections of known melanoma positive control. The standardized dilution was achieved as final titer in the cocktail by adding 20 μl MART-1, 100 μl Melan-A, and 200 μl Tyrosinase to 9.68 ml of DAKO Antibody diluent (Dako Corporation, Carpinteria, CA). ^¶ ^Adopted from Shidham et al [28].

## Material and methods

### Patients

We prospectively studied 51 lymph nodes (48 SLNs and 3 non-SLNs) from 25 patients (range- 1–4 SLNs per patient, mean- 2 per patient) under an IRB approved protocol at Froedtert Memorial Lutheran Hospital / Medical College of Wisconsin, Milwaukee, WI. A standard surgical protocol was used to identify the SLN [32]. The SLNs were harvested and submitted fresh to pathology for intraoperative and permanent section evaluation.

#### Pathologic Examination (Figure 1)

**Figure 1:**
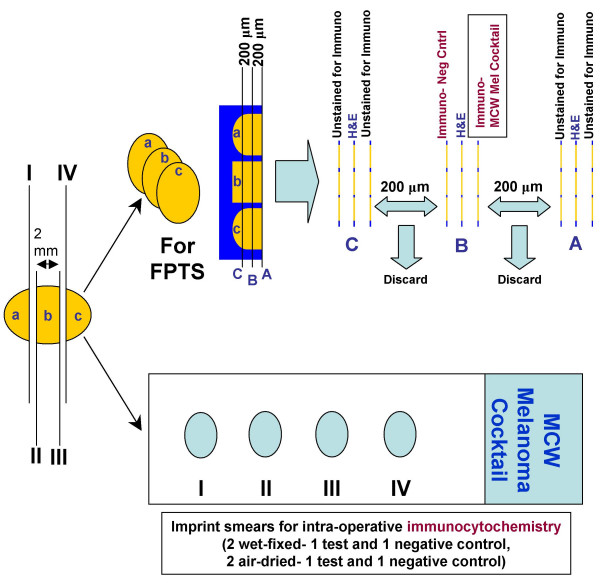
Pathological evaluation of sentinel lymph nodes for melanoma metastases. Section number 2, 5, & 8- stained with H & E; 4- immunostained with `MCW melanoma cocktail'; 6- negative control; 1, 3, 7, & 9- unstained. Number of slices of SLN shown (a,b,c) is just for illustration and would vary according to the size of the lymph node. (FPTS, formalin-fixed paraffin-embedded tissue sections; H & E, hematoxylin and eosin stain)

For the evaluation of the maximum surface area of the lymph node and most of the capsular area, the lymph nodes were transected perpendicular to the long axis as thin (not thicker than 2 mm) cross sections. Two pairs of imprint smears (one test and one negative control in each pair) were made by gently touching the cut surfaces to glass slides without allowing the imprint to be smeared. One of the pairs (1 test and 1 negative control) was air-dried, rehydrated in saline, and post-fixed in 'alcoholic formalin' [31] (see video clips as Additional file 1 Higher resolution- (for high speed connection); or Additional file 2 Low resolution- (for low speed connection), the screen shots in PDF file are available as Additional file 3  Screenshots). The other pair was wet-fixed by immersing the imprint smears in 95% ethanol before drying. Fixed smears were rinsed with 95% ethanol and then immunostained with 'MCW melanoma cocktail' (Table 1) using a rapid immunostaining protocol (Table 2). This rapid protocol required 17 minutes. Additional time required for smear preparation, smear processing, and evaluation of immunostained imprint smears may vary depending on the number of slides controlled by some variables such as the size and the number of SLNs submitted for evaluation.

**Table 2:** Rapid immunostaining protocols.

**Entirely manual**	**Partially manual and with Autostainer^†^**
1. Re-hydrate air- dried imprint smear with 0.9% saline- 15 seconds (slow~10 dips)	1. Re-hydrate air- dried imprint smear with 0.9% saline- 15 seconds (slow~10 dips)
2. Post-fix the re-hydrated smear in 'alcoholic formalin'*- 5 slow dips and then 1 minute	2. Post-fix the re-hydrated smear in 'alcoholic formalin'*- 5 slow dips and then 1 minute
3. Rinse the post-fixed smear with 95% ethanol: 5 dips	3. Rinse the post-fixed smear with 95% ethanol: 5 dips
4. Hydrate the smear in DW- 30 sec	4. 100% ethanol: 10 dips
5. 3% H_2_O_2 _in DW- 1 mt	5. 100% ethanol: 10 dips
6. Protein blocking solution- 1 mt	6. Methanol: 10 dips
7. 'MCW melanoma cocktail'**- 5 mt	7. 50%^¶ ^H_2_O_2_inMethanol: 1 mt with agitation
8. Rinse in 0.2% Tween 20 in DW	8. Deionized water: 10 dips
9. HRP-linker Antibody***- 5 mt	9. Tris buffer (ph 7.6): 10 dips
10. Rinse in tap water	10. Place smear on Dako Autostainer which automatically applies-
11. Chromogen (DAB)- 3 mt	a. Envision blocking Solution^††^- 1 mt
12. Rinse in tap water	b. 'MCW melanoma cocktail'**- 5 mt
13. Azure B (Blue solution of Diff-Quik^®^)- 1 mt	c. Envision+ Monoclonal HRP^†††^- 5 mt
14. Rinse in tap water	d. Chromogen (DAB)- 3 mt
15. Harris Hematoxylin- 30 sec	11. Remove the smear(s) and proceed with the following steps
16. Rinse in tap water	12. Deionized water: 10 dips
17. Dehydrate in ascending concentration of ethanol	13. Azure B- (Blue solution of Diff-Quik^®^)- 1 mt
18. Clear in xylene	14. Rinse in tap water
19. Coverslip the smear with the mounting medium	15. Harris Hematoxylin- 30 sec
	16. Rinse in tap water
	17. Dehydrate in ascending concentration of ethanol
	18. Clear in xylene
	19. Coverslip the smear with the mounting medium

mt, minute; sec, seconds; DW- Distilled water *Alcohol formalin was prepared by adding 50 ml of formalin (38–40% formaldehyde) to 350 ml of 95% ethanol and 100 ml of distilled water [modified and simplified from [31]; **'MCW melanoma cocktail'- Mixture of Melan- A, MART-1, & tyrosinase [28]; *** PowerVision™ Poly-AP anti-Mouse IgG (ImmunoVision Technologies, Co; Daly City, CA); ^†^DakoAutostainer; ^††^Dako Corporation, Carpinteria, CA; ^†††^Dako Envision+ (Dako Corporation, Carpinteria, CA); ^¶ ^3% 10 volume Hydrogen peroxide, USP (Hydrox Laboratories, Elgin, IL, USA). **Positive controls **were prepared from unfixed fresh melanoma tumor, which was immunoreactive for each of the individual components of the cocktail. The cut surface of the tumor was scraped with one end of glass slide and the scraped material accumulated at the end of the slide was spread between two glass slides similar to the spreading of bone marrow smears [32]. The air-dried smears were processed similar to 'test' slides. They were rehydrated in saline (10 to 20 seconds) and postfixed in alcoholic formalin (1 minute) [30]. The post-fixed smears were rinsed in 95% ethanol and then dehydrated by taking the smears through absolute ethanol, cleared in xylene, and cover-slipped with mounting medium. The cover-slip of positive control was removed by keeping the slide in xylene overnight (for this reason a formal communication to pathology and immunochemistry lab at least 24 hours before the SLN surgery is required). After removing the coverslip the smear was passed through absolute ethanol, 95% ethanol, and then joined with the protocol for immunostaining mentioned above. *These coverslipped smears of positive control could be archived at room temperature for long periods of time (personal experience). We have used such smears after removing the coverslip as positive controls up to 1 year later without loosing immunoreactivity for most of the commonly used immunomarkers.*

The first imprint smears from each pair were used as a 'test' and were immunostained with the 'MCW melanoma cocktail' by rapid protocol. The second imprint smear was used as a 'negative control' and processed in the same manner as the test slide except that Dako diluent^® ^was used in place of 'the cocktail'.

Numerous smears of positive controls (both air-dried and wet-fixed smears) were prepared previously from a melanoma tumor with a known immunoreactivity for each of the three components of 'the cocktail'. These smears were prepared by scraping the cut surface of the fresh melanoma tumor and spreading the scraped material between two slides as described previously [33]. The air-dried smears were fixed in alcoholic formalin after saline rehydration. Both smears (air-dried, saline rehydrated smears, post-fixed in alcoholic formalin and wet-smears fixed in 95% ethanol) were stored after processing them through ascending grades of alcohol and xylene, followed by mounting with a glass coverslip using mounting medium (Table 2).

Both air-dried and wet-fixed positive control smears were used during immunostaining for each batch of test smears by removing the coverslip following immersion of the slide in xylene for about 24 hours. This dissolves the mounting medium and separates the coverslip. After removal of the coverslip, the smears were put through absolute ethanol and then descending grades of ethanol to water to be combined with the respective step in the rapid immunostaining protocol (Table 2).

The immunostained imprint smears were evaluated for melanoma micrometastases. The test smears were compared with corresponding positive controls (air-dried versus wet-fixed). The wet-fixed test smear from a given SLN was compared with a respective air-dried test smear by evaluating the sharpness of immunostaining, morphological details of the immunostained tumor cells, frequency of staining of non-melanoma structures such as mast cells and erythrocytes, air-drying artifact, deterioration in the immunostaining of the cells with unequivocal features of tumor cells, and nonspecific background staining. The results were interpreted by pathologists as positive, indeterminate, or negative for melanoma metastases. For statistical analysis, indeterminate interpretations of immunostained imprint smears were considered negative. This was based on the clinical significance with reference to the intraoperative decision algorithm for the completion of regional lymphadenectomies in SLN positive cases.

After the preparation of imprint smears, the slices of SLNs were fixed in 10% formalin and processed for formalin-fixed paraffin-embedded tissue sectioning. These sections were evaluated according to the melanoma protocol (Figure 1) and immunostained by the avidin-biotin-peroxidase complex (ABC) method described previously [28].

## Results

**Figure 2:**
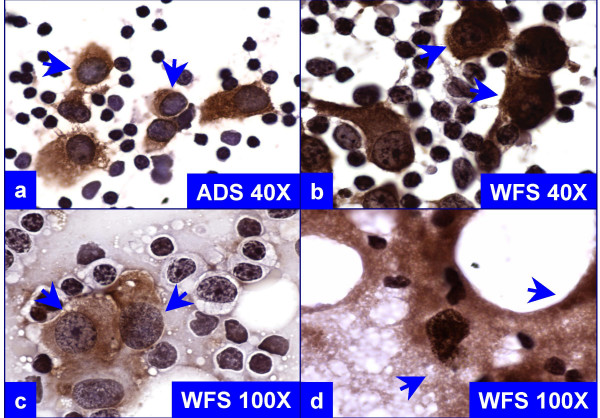
Comparison of cytomorphological features of immunostained, air-dried smears postfixed in alcoholic formalin after saline rehydration (ADS, 'a') versus wet-fixed smears fixed in 95% ethanol (WFS, 'b' through 'd'). The cytoplasmic immunostaining for the 'MCW melanoma cocktail' does not obscure the nuclei in 'a'. In contrast, immunostaining of shrunken cytoplasm around nuclei in wet-fixed smears obscures the nuclear details (arrows in 'b'). Air-drying artifact is present focally (arrows in 'c') with the presence of non-specific background staining (arrows in 'd').

Wet-fixed smears were difficult to prepare without focal air-drying artifact (Figure 2, Table 3). This difficulty was due to the time required to transfer each of the SLN slices on the glass slide one by one and then immersing the slide (with some of the imprints already dried) in 95% ethanol for wet fixation. This was not a concern while preparing the air-dried smears, as all the imprints were ultimately dried before processing (see Additional files 1,2,&3). The turnaround time for processing, immunostaining, and evaluating the smears was approximately 28 (range, 24–37) minutes.

**Table 3:** Comparison of air-dried versus wet-fixed imprint smears.

**S.No.**	**Feature**	**Air-dried imprint smears**	**Wet-fixed imprint smears**
1	Ease of preparing imprint smears of SLNs	Easy	Challenging
2	Air-drying artifact	Not applicable	Frequent
3	Non-specific background staining	Rare	Frequent
4	Immunostaining of non-melanoma structures	Rare	Common
5	Ease of processing, handling, and transporting the smears	Easy	Difficult
6	Loss of immunoreactivity of melanoma tumor cells due to air-drying artifact	Not applicable	Possible with potential for false negativity.
7	Sharpness of immunostaining	Present	Present
8	Shrinkage artifacts	Absent	Frequent
9	Morphological details of immunostained smears	Good	Poor
10	Potential loss of sample material on slide during immersion of slide in the fixative	Rare	Frequent

**Figure 3:**
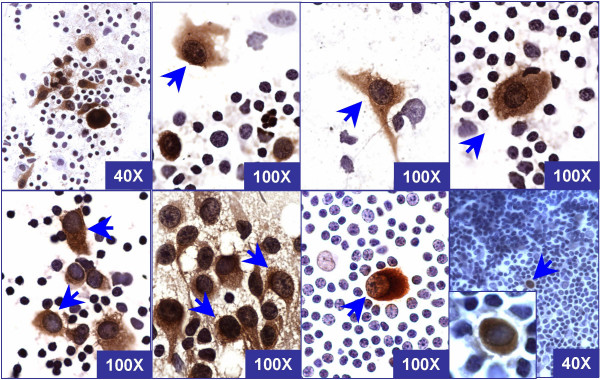
Cytomorphological spectrum of tumor cells (arrows) of melanoma metastases from different cases in rapidly immunostained air-dried imprint smears with the 'MCW melanoma cocktail' after saline rehydration and postfixation in alcoholic formalin. The tumor cells are large with well defined borders and show high nuclear to cytoplasmic ratio with non-granular cytoplasmic staining with clear nuclear details. The nuclear chromatin does not resemble the chromatin of adjacent lymphocytes in the background.

The immunostained tumor cells of melanoma metastases demonstrated a high nuclear/cytoplasmic ratio. The immunostaining was non-granular and cytoplasmic. The cytoplasmic immunostaining pattern facilitated the evaluation of the nuclear details. The cell margins were usually well defined. Unlike the chromatin of mast cells, the nuclear chromatin was not clumped and did not resemble the chromatin of the lymphocytes in the background. Nucleoli were usually prominent (Figure 3).

**Figure 4:**
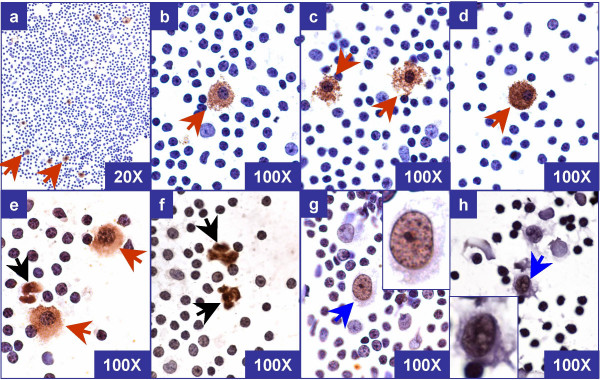
Morphological spectrum of *non-tumor structures *in rapidly immunostained air-dried imprint smears with 'MCW melanoma cocktail' after saline rehydration and postfixation in alcoholic formalin. a through e: Mast cells (brown arrows) show low nuclear/cytoplasmic ratio with granular staining of cytoplasm and fuzzy cell borders. The nuclear chromatin is clumped and resembled the chromatin of lymphocytes in the background. f: Non-nucleated ill defined structures (black arrow). g & h: Cells with immunoreactive nucleus (blue arrow). Insets of both g & h- zoomed cells with unequivocally negative cytoplasm but with brown staining of nucleus.

Because of the brief peroxidase blocking step, the endogenous peroxidase could not be blocked entirely in some cases, leading to the staining of some non-melanoma cells such as mast cells. These were detectable in both test smears and negative control smears. The mast cells showed smaller round nuclei with clumped chromatin. This clumped chromatin was comparable to the nuclear chromatin of adjacent lymphocytes in the background. The staining was coarsely granular. The cell margins of mast cells were usually hazy and ill-defined (Figure 4).

Rarely some nuclei demonstrated brown staining (the cocktail immunostaining is cytoplasmic and is not nuclear). The cells with such brown stained nuclei were morphologically consistent with histiocytes (Figure 4f &4g). Unequivocal nuclear staining without cytoplasmic immunostaining should be interpreted as negative in immunostained imprint smears. Brown non-nucleated round to irregular material (probably erythrocytes with unblocked endogenous peroxidase) was observed in a few cases (Figure 4h).

The non-specific staining was relatively frequent in wet-fixed smears (versus alcoholic formalin fixed saline rehydrated air-dried smears) and in manually immunostained smears (versus smears immunostained with Autostainer). These structures were usually interpreted as negative with ease. However, this factor could increase the interpretation time for negative cases due to the distraction effect and could prolong the crucial turn around time for intraoperative consultation.

Seventeen percent (8 out of 48) lymph nodes (28%, 7/25 cases) were positive for melanoma metastases in immunostained permanent sections. All melanoma metastases, except 1 SLN from 1 case, were demonstrated in both wet-fixed and air-dried imprint smears immunostained with the rapid protocol (sensitivity 89% and specificity 100%). On a case by case basis, 86% (6/7) of positive cases showed metastases in imprint smears immunostained with the 'MCW melanoma cocktail'; and demonstrated a sensitivity of 86%, a specificity of 100%, a negative predictive value of 95%, and a positive predictive value of 100%.

Imprint smears, immunostained with the rapid protocol, showed unequivocal melanoma metastases in 1 SLN which was negative by immunohistochemical evaluation of permanent sections. This was one of the SLN from a case with two other unequivocally positive SLNs in permanent sections and rapid immunostained imprint smears. This unequivocal positivity with immunostained imprint smears alone underscored the sampling benefit with imprint cytology.

Two SLNs (from 2 patients) interpreted as negative for melanoma metastases by immunohistochemical evaluation of permanent sections, were interpreted as indeterminate with the rapid protocol in both wet-fixed and air-dried smears. After retrospective evaluation, the rare doubtful cells observed in immunostained imprint smears were consistent with mast cells (Figure 4 a through 4e).

In 2 SLNs from 2 other cases, some scattered cells with non-granular immunostaining but with small, inconspicuous nuclei were observed. These cells were not mast cells and were absent in negative controls. They were also present as scattered single cells in permanent sections immunostained with 'the cocktail'. They were interpreted as benign and negative.

## Discussion

Metastases of melanoma tumor cells in SLN could be detected in imprint smears immunostained with the 'MCW melanoma cocktail'. The air-dried imprint smears from different SLNs were easier to prepare than wet-fixed smears. As reported previously, air-dried smears have numerous advantages [34]. Because of the shrinkage factor associated with wet-fixation, the cellular details are less distinct in wet-fixed smear as compared to air-dried smears. The wet-fixed smears frequently showed non-specific background staining. They also showed air-drying artifact, which frequently compromised the immunoreactivity, resulting in multifocal faint or false negative immunostaining (Table 3). Wet-fixed smears with a scant number of tumor cells may be translated into a false negative result because of air-drying artifacts.

Imprint smears (both air-dried and wet-fixed) immunostained with the 'MCW melanoma cocktail' showed excellent sensitivity and specificity (the indeterminate interpretations were equivalent to negative results). As compared to this, the alternative intraoperative approaches such as frozen-section alone [22], immunostaining of frozen sections with a cocktail of Melan- A, HMB-45, & tyrosinase [27], and the morphological evaluation of imprint smears alone [25,35] demonstrated relatively poor results. This is of practical significance. It facilitates the intraoperative decision to proceed with regional lymphadenectomy during the same anesthetic procedure.

Immunostained imprint smears unequivocally showed melanoma metastases in one SLN, but these melanoma metastases were not detected in permanent sections immunostained with 'the cocktail'. Two other SLNs from this case showed melanoma metastases in both immunostained permanent sections and imprint smears. This unequivocally positive result with immunostained imprint smears highlights the benefit of the enhanced sampling with imprint cytology.

Imprint smearing facilitates the sampling of two surfaces from each slice, except for the first and the last slice, as compared to only one surface of all slices by any sectioning method (Figure 1). In contrast to a sectioning method yielding 3–4 micron sections, which represent a tiny fraction of the lymph node slice, immunostained imprint smears facilitate the evaluation of the entire material sampled as an imprint on the glass slide from the cut surface of the lymph node.

The possibility of false positive results due to the cells of capsular nevi was disproved by the negativity of all immunostained imprint smears from 5 SLNs (5 cases) with capsular nevi. The cells in capsular melanocytic nevi located in the capsule and fibrous septa of lymph node did not exfoliate and adhere to slides during preparation of imprint smears. This appears to be due to the greater cohesiveness of the cells in capsular melanocytic nevi than the cells of malignant melanoma.

However, scattered cells with non-granular cytoplasmic brown staining which masked the small and inconspicuous nucleus were observed in 2 SLN of 2 cases. Such cells exhibiting benign morphology were also observed as scattered single cells in permanent sections immunostained with 'the cocktail'. These cells were interpreted as negative without significant challenge, but their exact nature could not be established. The possibility of singly scattered nevus cells was considered. Contrary to capsular nevus cells, such cells may be detached easily and picked up by the glass slide during the preparation of imprint smears. In some cases, they may cause an interpretation dilemma leading to indeterminate results even with permanent sections.

In 2 patients, 2 SLNs were interpreted as indeterminate with immunostained imprint smears. They were interpreted as negative by the immunohistochemical evaluation of permanent sections. Cytomorphologically, the rare doubtful cells present in immunostained imprint smears were consistent with mast cells. They were also present in the respective negative controls (Figure 4).

As endogenous peroxidase activity could not be blocked completely during the short endogenous peroxidase blocking step in the rapid protocol, non-melanoma cells such as mast cells may show brown staining in some cases. Familiarity with the morphological spectrum of immunostained tumor cells (Figure 3) and other non-specifically stained cells including mast cells (Figure 4) in immunostained imprint smears should prevent the indeterminate interpretation of these cells in future.

The positive control smears may be prepared from time to time utilizing fresh, unfixed melanoma tumors for long term availability. Alternatively, the smears of melanoma tumor cell lines immunoreactive to individual components of the cocktail may be used after processing and fixing similar to test smears. These smears may be dehydrated and coverslipped (Table 2). Coverslipped positive control smears could be archived at room temperature for extended time periods. Coverslips can be removed by immersing the slides in xylene (usually 24 hours) to dissolve the mounting medium and to loosen the glass coverslip from the slide. We have used such smears after removing the coverslip as positive controls up to 1 year after they were originally prepared without affecting immunoreactivity for most of the commonly used immunomarkers including the 'MCW melanoma cocktail' (personal experience). Since a positive control had to be processed in advance by removing the coverslip in xylene, a notice at least one day prior to the intraoperative evaluation was required routinely as a part of the protocol.

Imprint smears are easy and quick to make without incurring significant expense. They are faster than frozen sectioning and help prevent the loss of tissue associated with cryosectioning. Frozen-sectioning of lymph nodes is frequently problematic because of fat, either adjacent to or in the lymph node. These problems are circumvented with imprint smears, which would also prevent problems associated with the interpretation of final permanent sections of frozen tissue.

For billing and reimbursement purpose, the rapid intraoperative evaluation of immunostained imprint smears may be coded with existing CPT (Current Procedural Terminology) codes- 88329 for the intraoperative consultation, 88161 for the preparation and processing of the imprint smears, and 88342 for the immunostaining of the imprint smears with interpretation [36].

As a future prospect, a 'cocktail' of directly conjugated individual antibodies (with a peroxidase or similar indicator system) used for a one step immunostaining method resulting in a significant reduction in immunostaining time (up to 6 minutes) with fewer staining steps would simplify the procedure [37]. As discussed above, the rapid protocol may not block the endogenous peroxidase. Rapid blocking of endogenous peroxidase with specific inhibitors / blocking agents, without affecting the cytomorphology, could prevent the non-specific staining of mast cells. This would improve the interpretation speed and confidence by eliminating the distraction factor of non-specifically stained cells thus reducing the chances of indeterminate interpretations and simplify the learning curve.

In summary, air-dried imprint smears which were postfixed in alcoholic formalin following saline rehydration were optimal for immunocytochemical evaluation with the 'MCW melanoma cocktail'. Wet-fixed smears did not compromise the immunoreactivity of 'the cocktail', but they were difficult to prepare without air drying artifact and non-specific background staining. Capsular melanocytic nevi did not show false positive results. The rapid evaluation of imprint smears immunostained with the 'MCW melanoma cocktail' is reliable for the intraoperative evaluation of cutaneous melanoma SLNs for melanoma metastases.

## List of abbreviations

ABC, avidin-biotin-peroxidase complex; DAB, Diaminobenzidine Hydrochloride; H&E, hematoxylin and eosin; MCW, Medical College of Wisconsin; SLNs, Sentinel Lymph Nodes.

## Competing interests

None.

## Authors' contributions

VS conceived, designed, carried out the entire study in addition to the standardization of MCW melanoma cocktail protocol and preparation of manuscript. RK participated in its design and coordination. GD performed the immunohistochemical staining including standardization of the MCW melanoma cocktail with VS and SK. WD organized the clinical participation including recruiting of cases and arranging patients consent. All authors read and approved the final manuscript.

## Supplementary Material

Additional file 1 Higher resolution - (for high speed connection) Click here for file

Additional file 2 Lower resolution - (for low speed connection) Click here for file

Additional file 3 Screen shots Click here for file
