# Supplementary material for: Optimization of an immunostaining protocol for the rapid intraoperative evaluation of melanoma sentinel lymph node imprint smears with the 'MCW melanoma cocktail'
Source: Cytojournal. 2004 Aug 6;1:2. doi: 10.1186/1742-6413-1-2 (PMC524024; doi:10.1186/1742-6413-1-2)
Supplement: Additional file 3 — Screen shots [file 1742-6413-1-2-S3.pdf]

Preparation of Imprint Smears

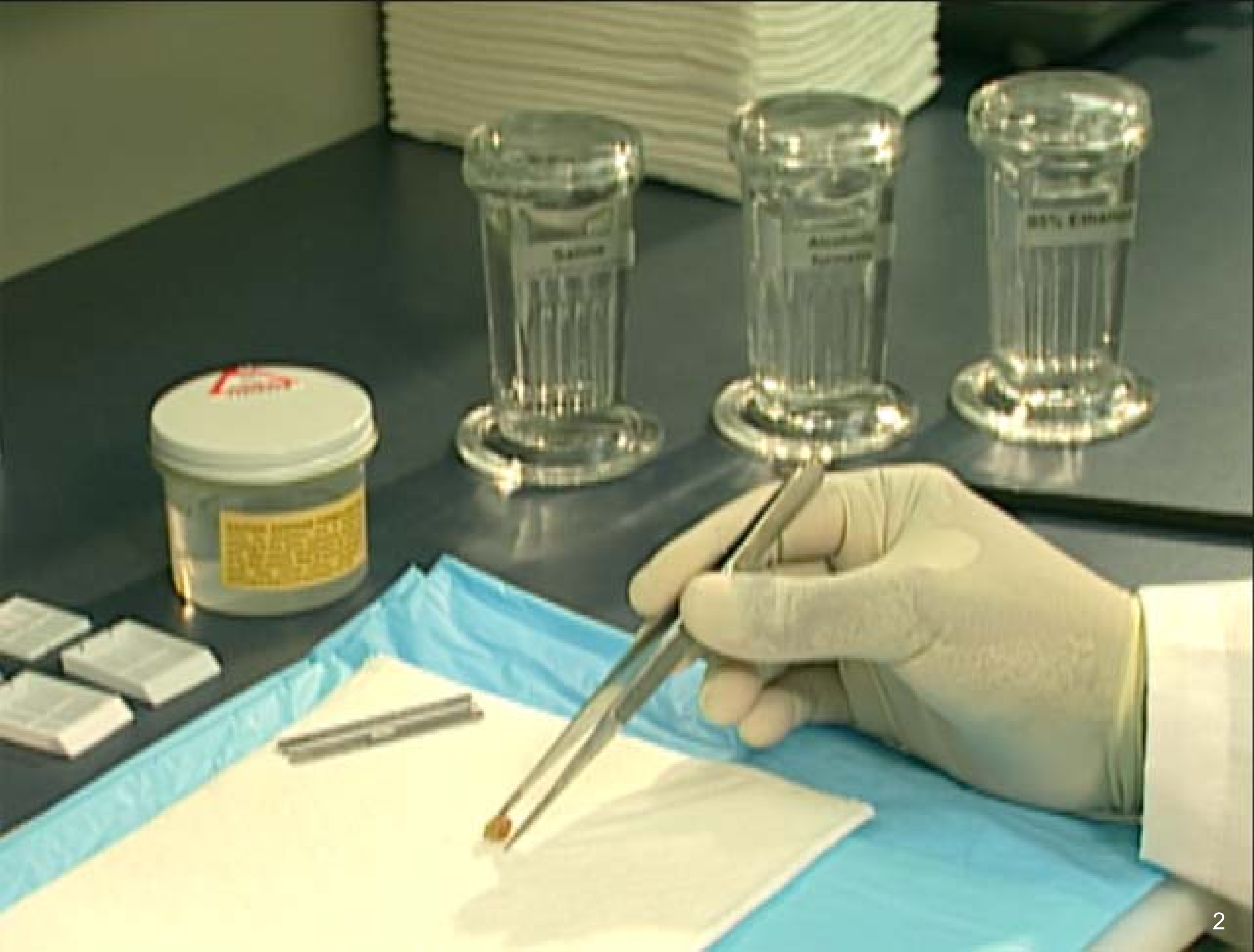

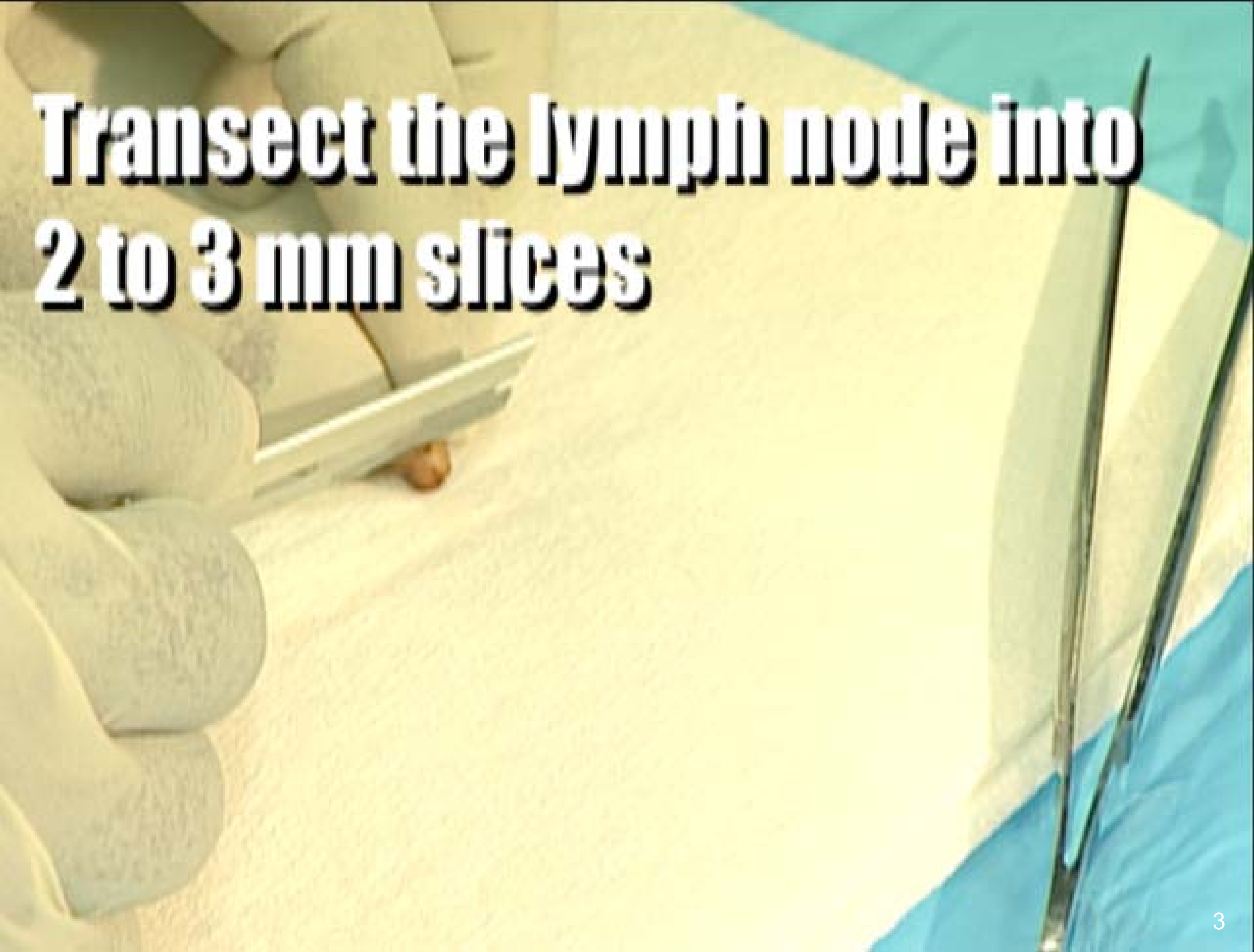
Transect the lymph node into
2 to 3 mm slices

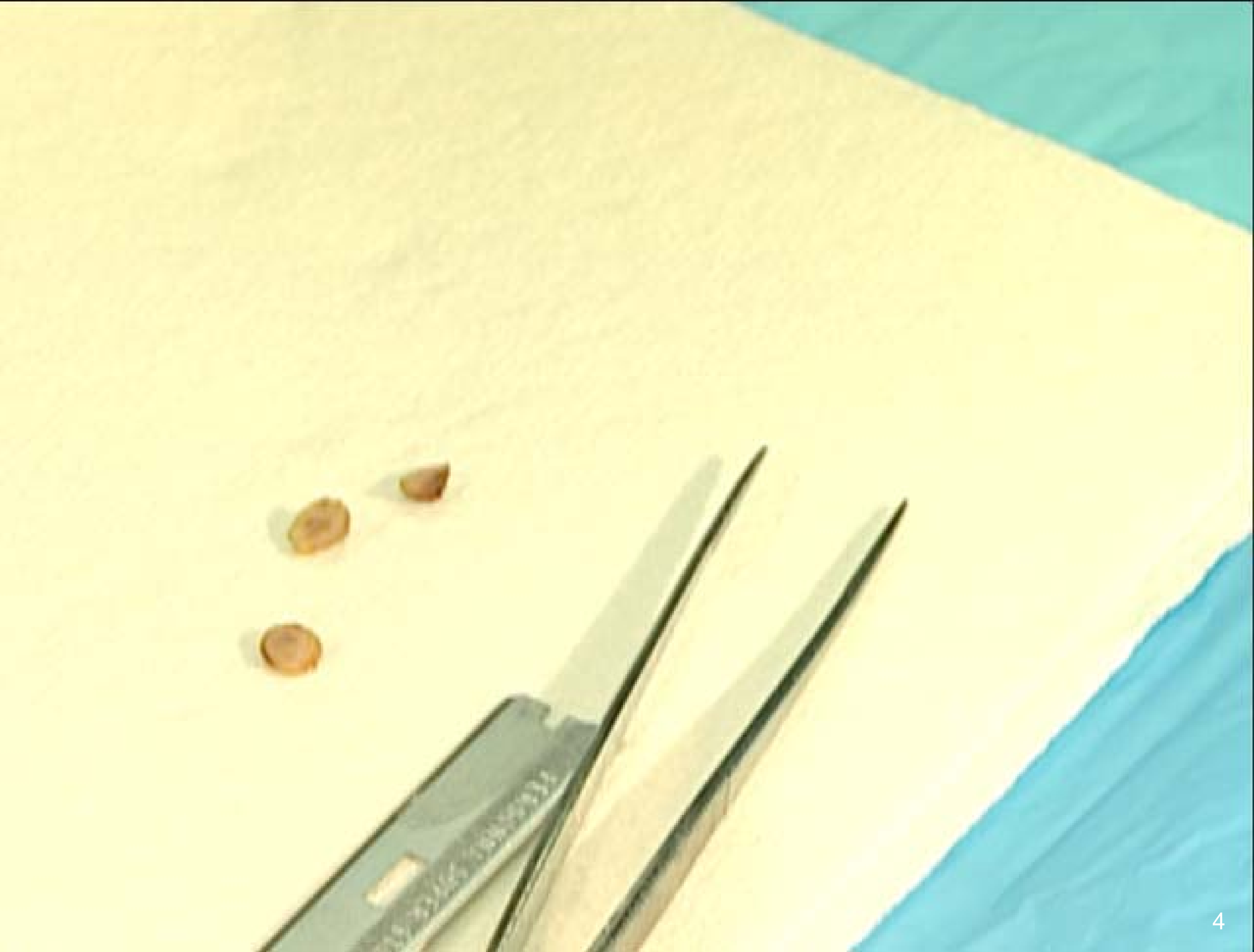

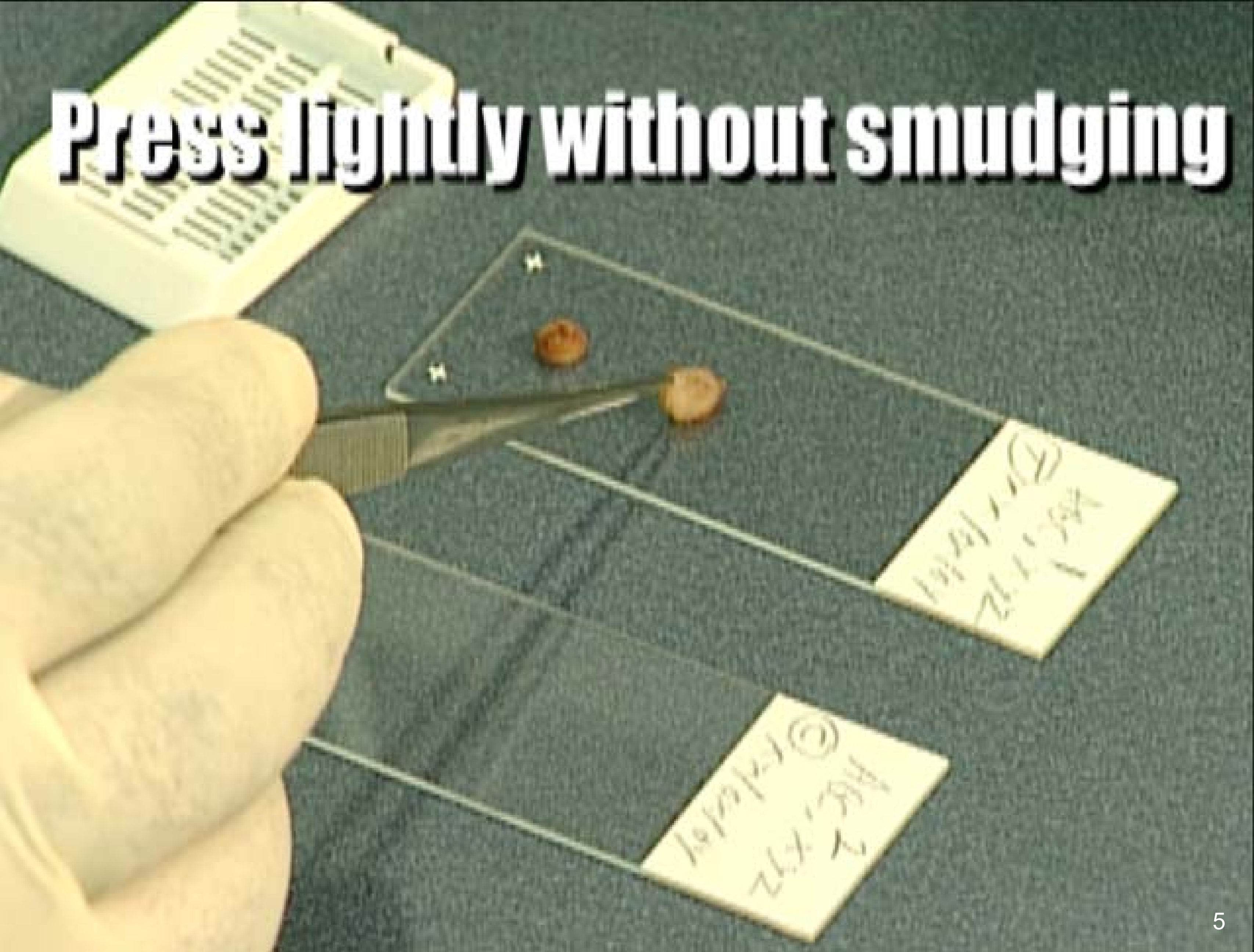
Press lightly without smudging

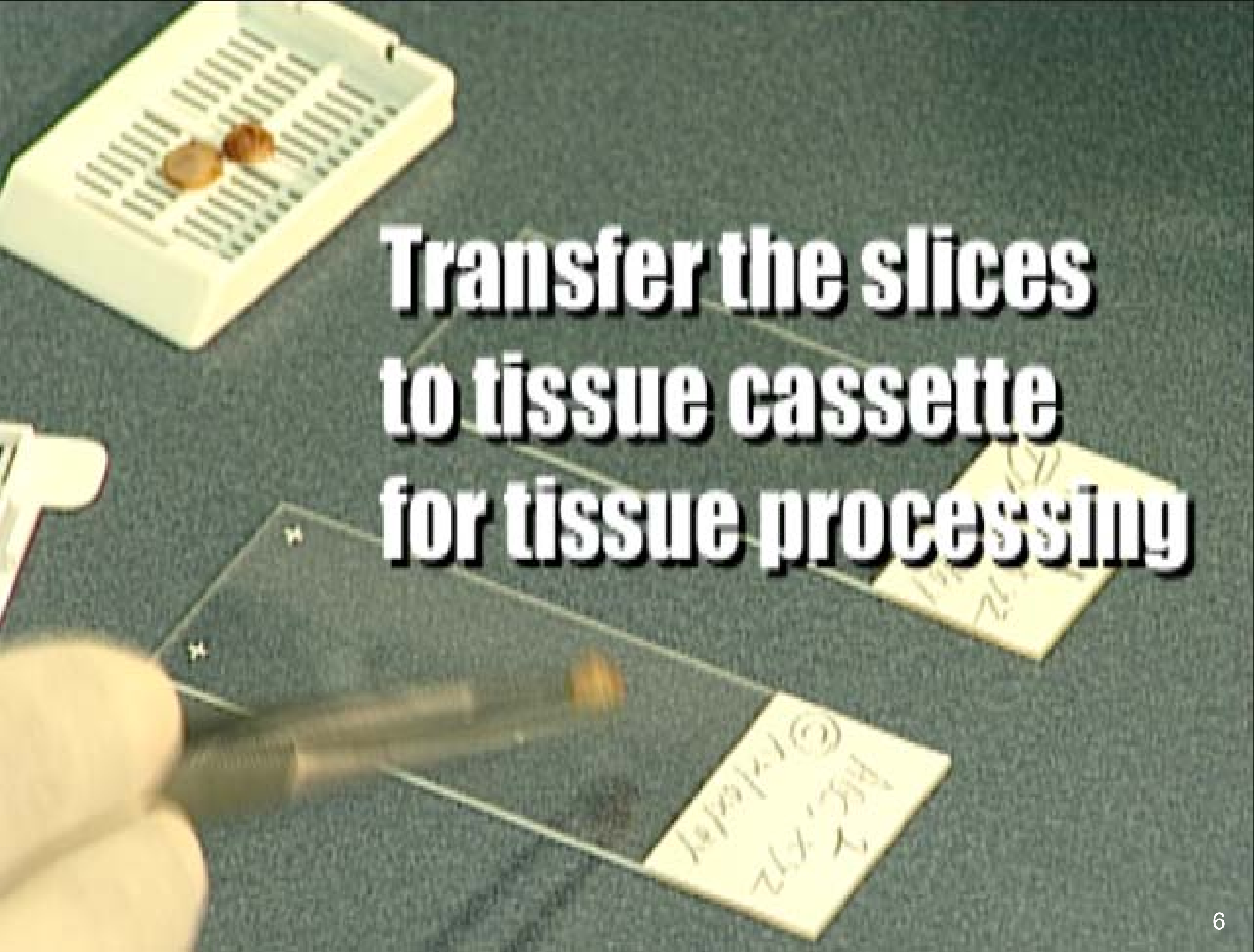
Transfer the slices
to tissue cassette
for tissue processing

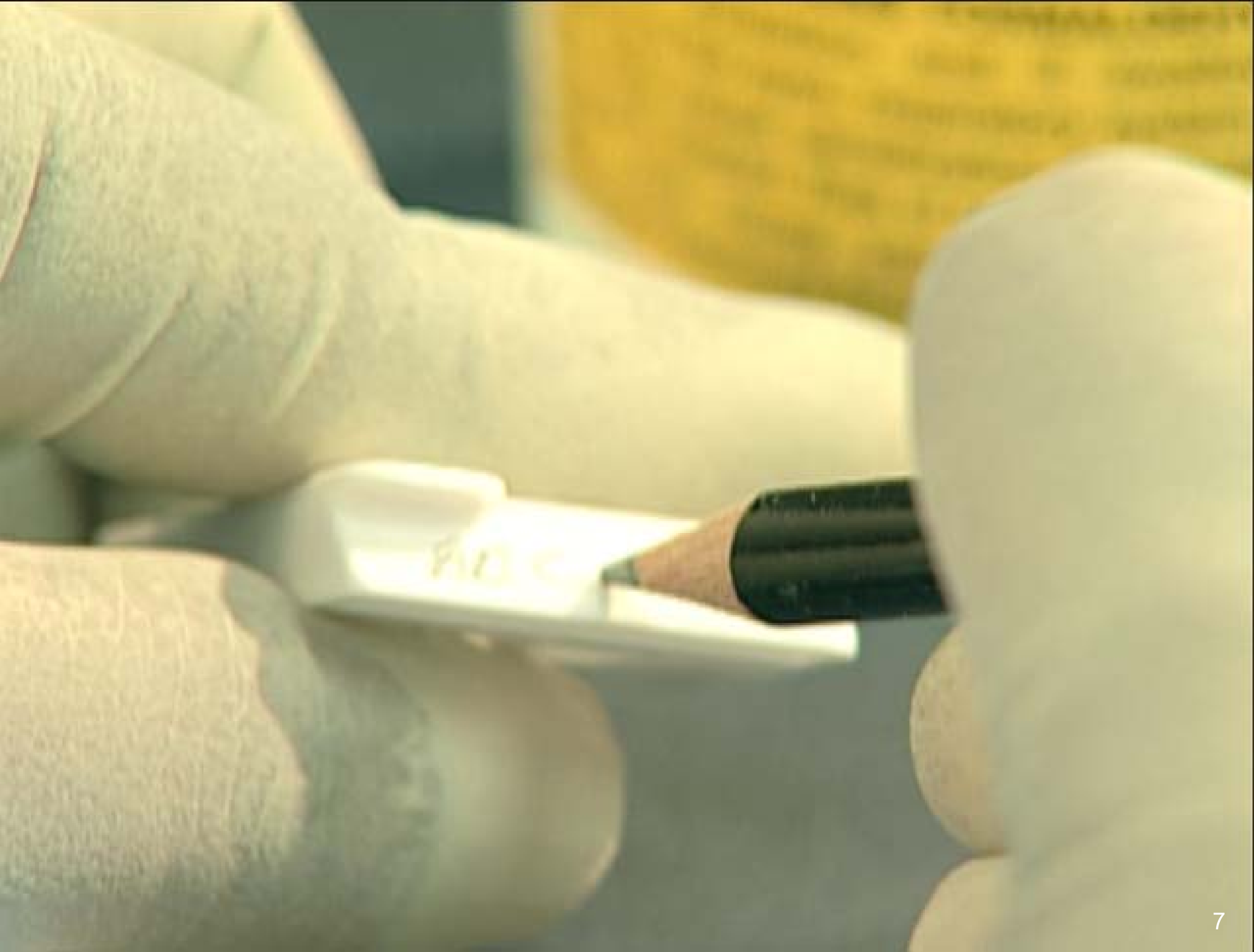

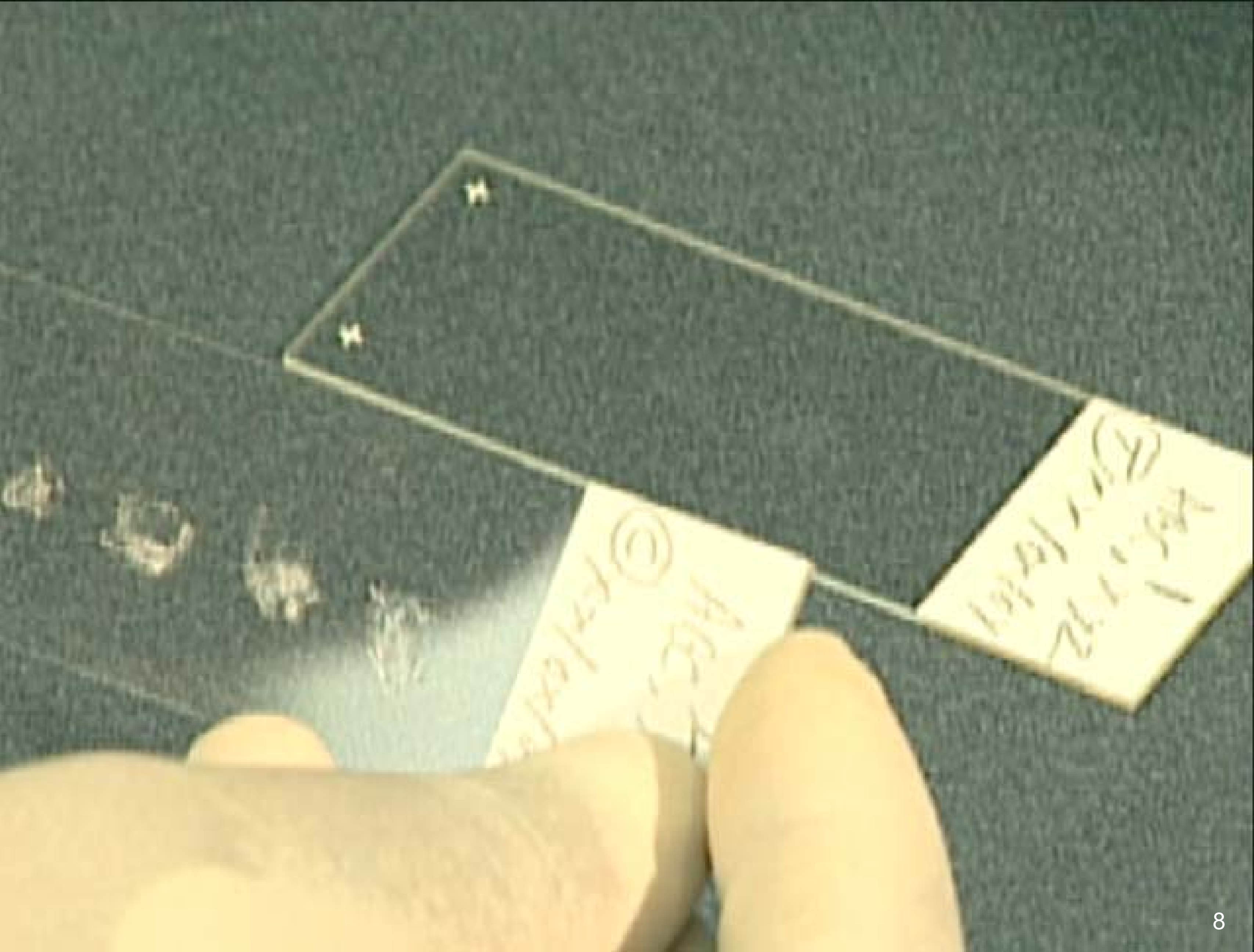

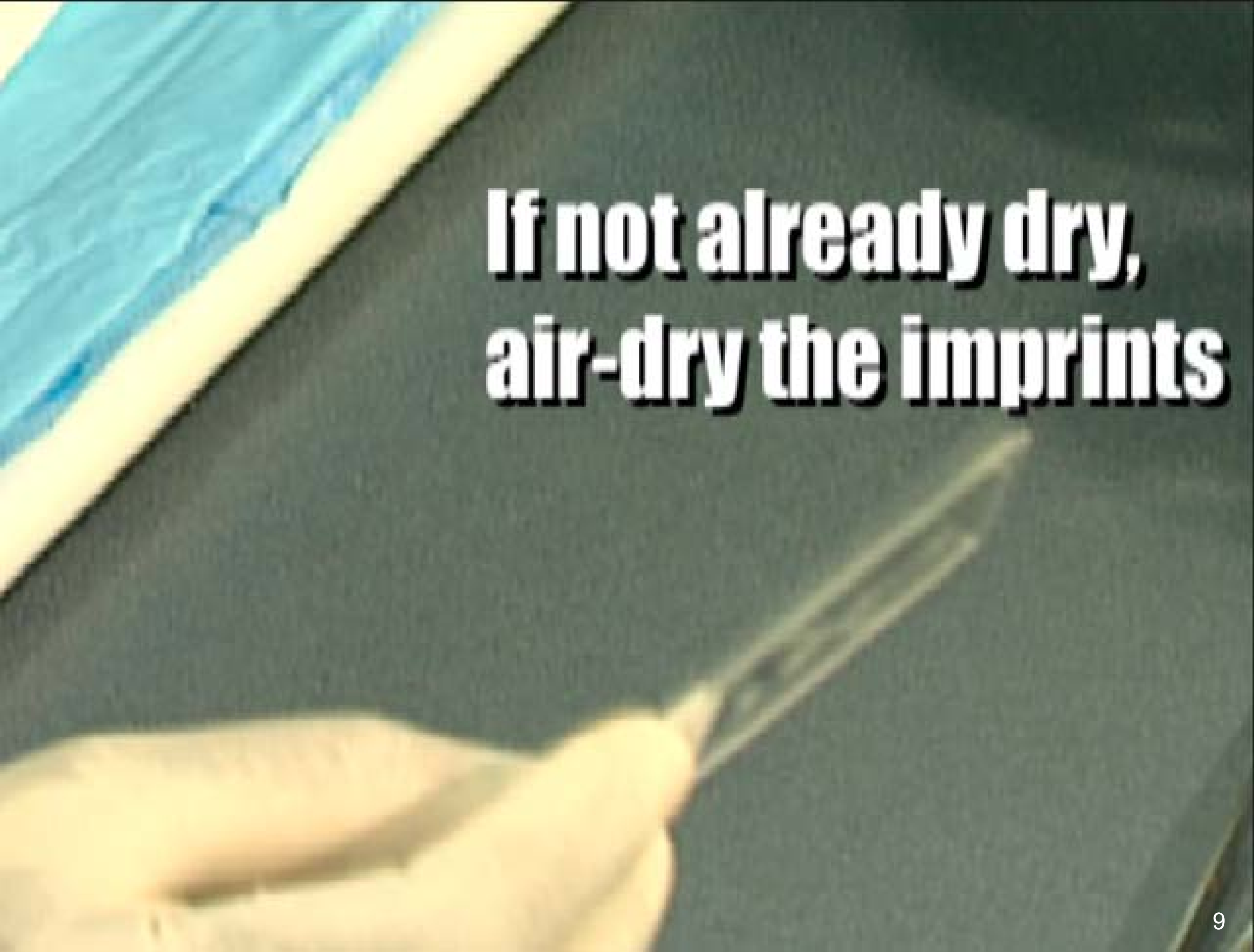
If not already dry,
air-dry the imprints

Rehydrate in 0.9% saline (15 seconds, 10 slow dips)

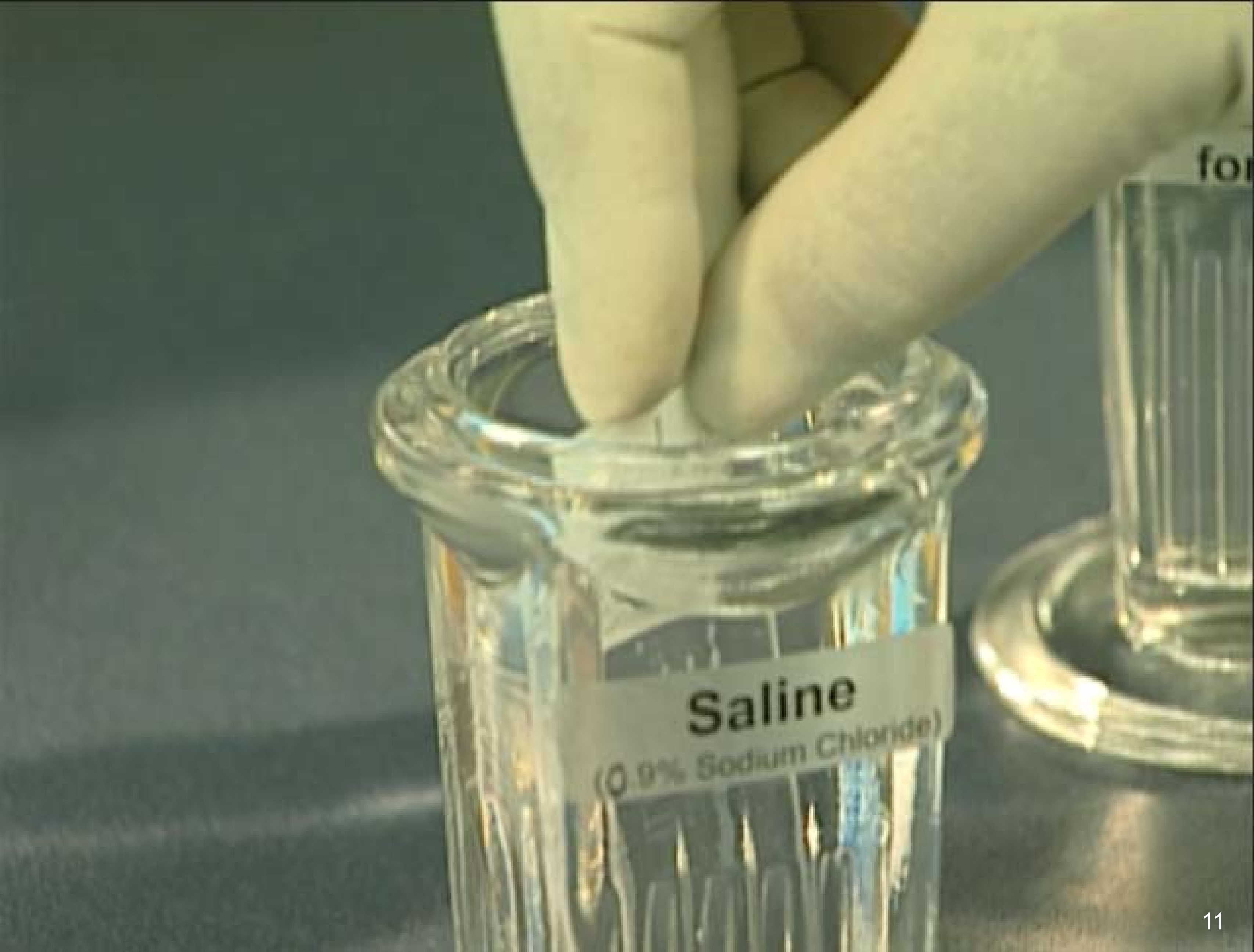
Saline
(0.9% Sodium Chloride)

Post-fix in alcoholic formalin

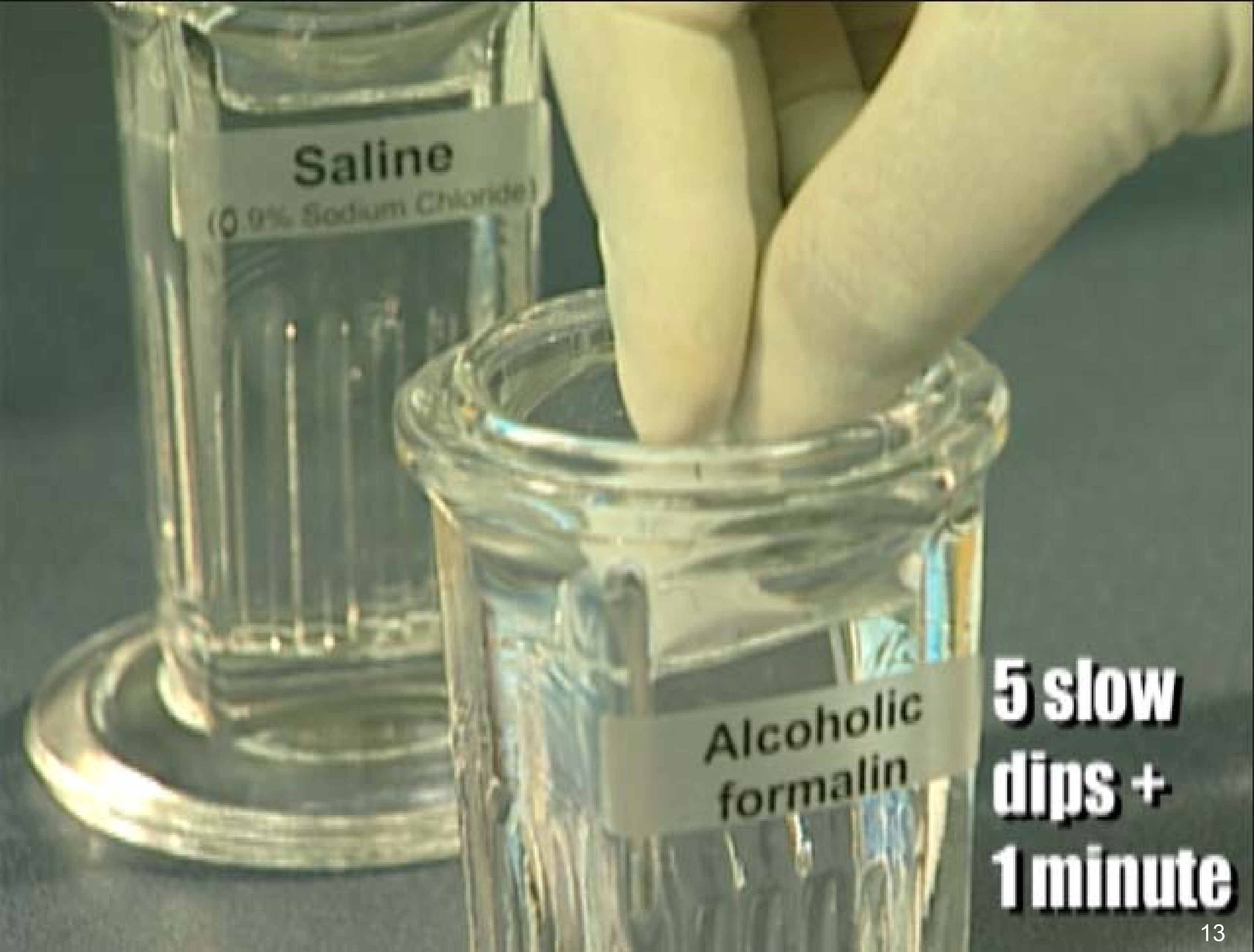
Saline
(0.9% Sodium Chloride)
Alcoholic
formalin
5 slow
dips +
1 minute

Rinse with 95% ethanol
(5 dips)

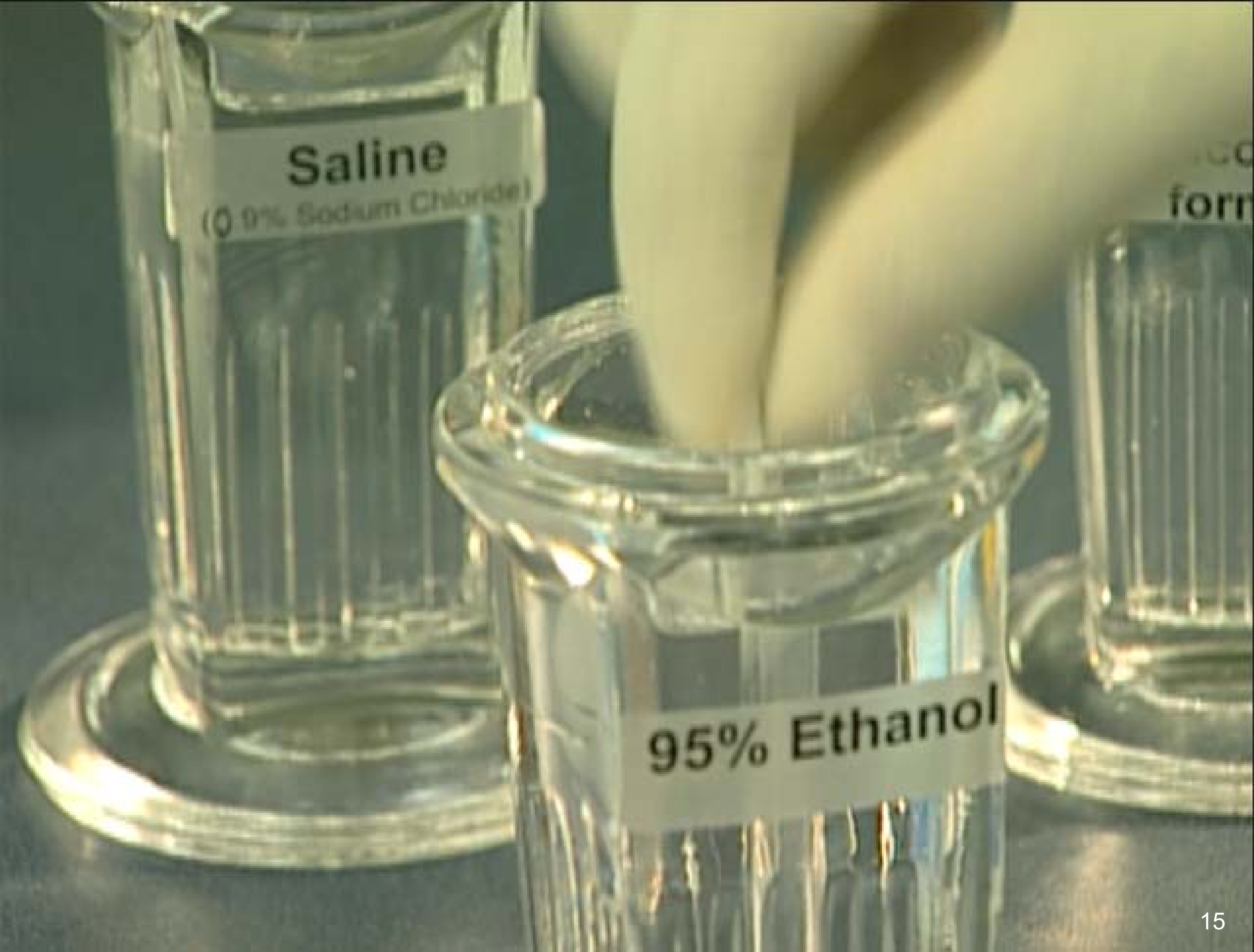
Saline
(0.9% Sodium Chloride)
95% Ethanol

Proceed with rapid immunostaining protocol
